# Modification of Transfer RNA Levels Affects Cyclin Aggregation and the Correct Duplication of Yeast Cells

**DOI:** 10.3389/fmicb.2020.607693

**Published:** 2021-01-15

**Authors:** Loreto Arias, Fabián Martínez, Daniela González, Rodrigo Flores-Ríos, Assaf Katz, Mario Tello, Sandra Moreira, Omar Orellana

**Affiliations:** ^1^Programa de Biología Celular y Molecular, Instituto de Ciencias Biomédicas, Facultad de Medicina, Universidad de Chile, Santiago, Chile; ^2^Departamento de Biología, Facultad de Química y Biología, Universidad de Santiago de Chile, Santiago, Chile

**Keywords:** transfer RNA, cell cycle, cyclin, codon usage, protein aggregation

## Abstract

Codon usage bias (the preferential use of certain synonymous codons (optimal) over others is found at the organism level (intergenomic) within specific genomes (intragenomic) and even in certain genes. Whether it is the result of genetic drift due to GC/AT content and/or natural selection is a topic of intense debate. Preferential codons are mostly found in genes encoding highly-expressed proteins, while lowly-expressed proteins usually contain a high proportion of rare (lowly-represented) codons. While optimal codons are decoded by highly expressed tRNAs, rare codons are usually decoded by lowly-represented tRNAs. Whether rare codons play a role in controlling the expression of lowly- or temporarily-expressed proteins is an open question. In this work we approached this question using two strategies, either by replacing rare glycine codons with optimal counterparts in the gene that encodes the cell cycle protein Cdc13, or by overexpression the tRNA^*Gly*^ that decodes rare codons from the fission yeast, *Schizosaccharomyces pombe*. While the replacement of synonymous codons severely affected cell growth, increasing tRNA levels affected the aggregation status of Cdc13 and cell division. These lead us to think that rare codons in lowly-expressed cyclin proteins are crucial for cell division, and that the overexpression of tRNA that decodes rare codons affects the expression of proteins containing these rare codons. These codons may be the result of the natural selection of codons in genes that encode lowly-expressed proteins.

## Introduction

Degeneracy or redundancy of the genetic code implies that more than one codon (2, 4 or 6 codons) exist for 18 of the 20 genetically encoded amino acids. Codons encoding the same amino acid are called synonymous. Despite considerable information demonstrating that the choice of one synonymous codon over another is not random (Bulmer, 1991; reviewed in Quax et al., 2015), the term “silent” codons is still in use, since codon exchange does not alter protein sequences. Each organism has a defined codon usage bias (CUB). CUB greatly varies among species and within the same genome.

Whether CUB is the result of mutational drift forced by the nucleotide composition of DNA and/or natural selection for translation efficiency or accuracy has been a matter of debate (Bulmer, 1991; Brandis and Hughes, 2016; Dilucca et al., 2018). The most frequent codons (optimal codons) in rapidly-growing unicellular organisms are usually decoded by highly-expressed tRNAs, and the less frequent codons (rare or non-optimal codons) are decoded by less-expressed tRNAs (Ikemura, 1985; Kanaya et al., 1999; Chaney and Clark, 2015; Quax et al., 2015). In these organisms, CUB may optimize or deoptimize translation (translation efficiency) for a group of related mRNAs, coordinating their expression. However, these interpretations are controversial since some researchers have established that codon usage is an important factor in protein expression in trypanosomatids (Jeacock et al., 2018), where protein concentration can be estimated from protein coding sequences, while in other studies it has been determined that translation elongation speed is independent of codon usage bias (Ingolia et al., 2011). Moreover, codon bias has been identified as a major factor in determining both mRNA (Presnyak et al., 2015) and protein levels (Zhou et al., 2016). However, the extent to which mRNA translation efficiency links codon bias to protein levels remains unclear. This issue has gained importance in recent years as it has been linked to many factors that affect gene expression, such as mRNA stability, protein levels, folding, and localization (Chaney and Clark, 2015; reviewed in Bali and Bebok, 2015; Buhr et al., 2016; Hanson and Coller, 2018). Among these factors, the effect of codon choice on codon reading speed is the least understood. Since protein folding is co-translational for many proteins, codon changes that affect codon reading speed may also alter the folding of encoded proteins, leading to abnormal protein functioning. Experimental evidence and genome-wide analyses suggest that regions between protein domains are enriched in non-optimal codons, while structured domains are mostly encoded by optimal codons (Zhou et al., 2015). Despite these data, it is difficult to predict the synonymous mutations that lead to abnormal protein expression, folding, or function and the response of cells to these defects.

Lowly expressed proteins have a tendency to use rare codons. Even though there is a correlation between gene expression and rare codon use in various species (Hiraoka et al., 2009; Ray et al., 2014), it is still not clear if the tendency to use non-optimal codons plays a role in protein expression levels or in protein folding (Supek, 2016).

Other aspects of genetic information may be altered by the replacement of synonymous codons. It is relevant to consider these potential effects since cell function can be altered. It has been proposed that codons are selected to prevent off-frame translation after ribosomal slippage (ambush hypothesis) (Seligmann and Pollock, 2004; Seligmann, 2010, 2012, 2019; Køížek and Køížek, 2012), although this idea has been disputed (Morgens et al., 2013; Chatenay et al., 2017). Additionally, circular codes, that is, codes within the genetic code, have been proposed as playing a role in ensuring translation accuracy (Arquès and Michel, 1996). Proposed as primordial codes, they are conserved in evolution, with implications in the interaction of mRNAs with ribosomes and tRNAs (Michel and Thompson, 2020). Codon bias may be implicated in the conservation of circular codes in organisms and replacement of synonymous codons may alter such codes.

Circadian-clock proteins of *Neurospora crassa* and a cyanobacterium are examples of how the replacement of non-optimal codons with optimal ones affects the function of encoded proteins (Xu et al., 2013; Zhou et al., 2013) and alters circadian rhythms. A close correlation between the translation of non-optimal codons and the level of decoding, tRNA has been revealed in the gene encoding the cystic fibrosis transmembrane conductance regulator (CFTR). The replacement of an optimal with a non-optimal codon affects the function of CFTR. This effect is compensated by the upregulation of tRNA decoding non-optimal codon (Kirchner et al., 2017). Based on the above mentioned, the classical role of tRNAs as adaptor molecules for the incorporation of amino acids in nascent proteins has been expanded by findings that demonstrate changes in global tRNA abundance in response to different cellular processes (Torrent et al., 2018; Yang et al., 2020).

Eukaryotic cell-cycle proteins (cyclins) appear to be enriched in non-optimal codons, with some differences that depend on the cell-cycle phase in which they are expressed. The levels of total tRNA and of some aminoacyl-tRNA synthetases are also cell-cycle dependent in yeast (Frenkel-Morgenstern et al., 2012). Cdc13 is one of the most widely studied cyclins from the fission yeast, *S. pombe*. It forms a complex and activates Cdc2 (Cdk1), an important kinase in G2/M transition (Humaidan et al., 2018). Cdc13 is preferentially expressed during this transition. The complex travels to the nucleus and phosphorylates a number of substrates that are crucial for the progress of the cell cycle. Although the protein structure of Cdc13 has not yet been determined, a secondary structure is predicted, and some relevant regions have been identified. Cdc13 has a conserved hydrophobic patch (MRGILTDW) that is not required for cells to undergo the S phase *in vivo* but is required to target Cdc13 to the equivalent to the spindle pole body in yeast (SPB) and for mitosis (Basu et al., 2020). Mutation in this hydrophobic patch alters Cdc13 localization, preventing centrosomal localization at the onset of mitosis. Finally, the complex is degraded at the mitosis stage. Since *cdc13* contains several non-optimal codons, we hypothesize that the presence of non-optimal codons in *cdc13* is required to modulate the proper level of the Cdc13 protein. The variable levels of tRNAs during the cell cycle (Frenkel-Morgenstern et al., 2012) may control the expression of *cdc13*. To test this hypothesis, we followed two approaches: (1) to introduce synonymous mutations to replace non-optimal with optimal codons in *cdc13*, and (2), to modify the concentration of tRNAs that decode non-optimal codons. Our results show that these alterations significantly affect both Cdc13 distribution in soluble and aggregated fractions and cell duplication.

## Materials and Methods

### Yeast Strains and Media

*Schizosaccharomyces pombe* 972h-Sleu1-32 (LP36) strain was used in homologous recombination experiments. Supplementary Table 1 lists the primers used in this study. *S. pombe* was grown on YES medium (5 g/l of yeast extract, 30 g/l of glucose), YPD (10 g/l of yeast extract, 20 g/l of peptone, 20 g/l of glucose), and Edinburgh minimal medium 2 (EMM2, United States Biological).

### Plasmid Construction for Homologous *cdc13* Recombination

The endogenous *cdc13* gene was replaced by a homologous recombination with the wild-type or mutated *cdc13*, both containing a 7xHis-tag at the 3′ end. For the homologous recombination with the wild-type *cdc13* gene, we first cloned the flanking regions of the Cdc13 coding sequence in the pFA6a-KanMX6 vector (Addgene), as described below. Flanking regions were amplified from *S. pombe* genomic DNA (gDNA), using Herculase II Fusion DNA polymerase (Agilent Genomics) in accordance with the manufacturer’s instructions, using cdc13-histag forward and reverse primer sets as described in Supplementary Table 1. The digestion products were ligated to the pFA6a-KanMX6 vector (first the 3′ flanking region, followed by the 5′ flanking region), previously digested with the corresponding restriction enzymes (*Sac*I and *Eco*RI to 3′ flanking region; *Nde*I and *Bam*HI to 5′ flanking region). The *E. coli* JM109 strain was transformed with the corresponding construction by chemical transformation (Sambrook and Russell, 2001; Froger and Hall, 2007) and five clones were analyzed by DNA sequencing to corroborate the correct incorporation of 7xHis-tag. The final vector was named pFA6a-KanMX6-5′-3′ and confirmed by DNA sequencing.

The synonymous substitutions were designed based on *S. pombe* codon usage (Forsburg, 1994; Hiraoka et al., 2009) replacing low-usage codons GGG/GGA (five codons) with their optimal counterpart GGT. Supplementary Table 1 shows the primers that were used. *Cdc13* mutants were constructed according to the following methodology: first, the mutant *cdc13* sequence was amplified in five separate PCRs, using the following primers listed in Supplementary Table 1: cdc13-histag-F and CDC13_GGT1_R (reverse primer containing the first mutated codon) were used to amplify the first segment; CDC13_GGT1_F and CDC13_GGT2_R (reverse primer containing the second mutated codon) were used to amplify the second segment; CDC13_GGT2_F and CDC13_GGT3_R (reverse primer containing the third mutated codon) were used to amplify the third segment; CDC13_GGT3_F and CDC13_GGT45_R (reverse primer containing the last two mutated codons) were used to amplify the fourth segment; and CDC13_GGT45_F forward primer containing the last two mutated codons) and cdc13-histag-R were used to amplify the last segment. All PCR products were purified using the Real Genomics HiYieldTM Gel/PCR DNA fragments Extraction system commercial kit (Real Genomics) in accordance with the manufacturer’s instructions, and then the purified PCR products were used as templates for four amplifications by PCR: segments I and II were used as templates to join both segments, amplifying with cdc13-histag-F and CDC13_GGT2_R primers. In another experiment, segments III and IV were used as templates to join both segments, amplifying with CDC13_GGT2_F and CDC13_GGT45_R primers (Supplementary Table 1). Both PCR products were purified the same way and were used as templates to join the four segments by amplified PCR with cdc13-histag-F and CDC13_GGT45R primers (Supplementary Table 1). This product was joined with fragment V as described previously, amplified with cdc13 histag-F and cdc13-histag-R primer sets (Supplementary Table 1), resulting in the complete *cdc13* with the mutations. The final product was purified and digested with *Nde*I and *Bam*HI restriction enzymes, and then ligated to the pFA6a-KanMX6-5′-3′ vector. The product was transformed into the *E. coli* JM109 strain by chemical transformation (Sambrook and Russell, 2001; Froger and Hall, 2007) and 5 clones were analyzed by DNA sequencing to corroborate the correct incorporation of the mutations.

### Homologous Recombination

Purified plasmids containing the flanking regions of the wild-type plus mutant coding sequences of Cdc13 and the His-tag were used to amplify the sequence used for homologous recombination (Oldenburg et al., 1997), using Herculase II Fusion DNA polymerase (Agilent Genomics) in accordance with the manufacturer’s instructions, and using pREP41F′ and pREP41R′ primers (Supplementary Table 1). 200 ng of the corresponding PCR product and 300 ng of the pET15b vector (as a carrier) were electroporated in 100 μl of electrocompetent *S. pombe* cells were prepared with the described protocol (Forsburg and Rhind, 2006). Yeasts were washed with 1 M sorbitol and recovered in YPD medium at 30°C for 3 h. The cells were then pelleted, resuspended in 200 μl of YE medium, and plated in YPD agar prepared with 200 μg/ml of G418 (geneticin) antibiotic (Sigma-Aldrich). Cells were grown for 4–5 days at 30°C and single colonies were picked and grown on YPD medium supplemented with 200 μg/ml of G418 antibiotic. The incorporation of synonymous mutations at the right position was corroborated by DNA sequencing.

### Overexpression of tRNAs

Fragments containing tRNA genes (tRNA^*Gly*^_*UCC*_, tRNA^*Gly*^_*GCC*_, and tRNA^*Arg*^_*UCC*_) were amplified by PCR from *S. pombe* genomic DNA using Herculase II Fusion DNA polymerase (Agilent Genomics) in accordance with the manufacturer’s instructions and using the primer set described in Supplementary Table 1. The PCR products contained 400 base pairs for tRNA^*Gly*^_*UCC*_ and 420 base pairs for tRNA^*Gly*^_*GCC*_ and tRNA^*Arg*^_*UCC*_, including the regulatory elements necessary for tRNA transcription. The products were purified using the commercial kit Real Genomics HiYieldTM Gel/PCR DNA Fragment Extraction (Real Genomics), in accordance with the manufacturer’s instructions. The purified products were digested with *Bam*HI and *Nde*I and cloned into pREP41, a high copy number vector. Vectors with tRNA genes were transformed into yeast by electroporation, as described previously (Forsburg and Rhind, 2006).

### Northern Blot Analyses

Northern blot analysis was performed using biotinylated probes synthesized by IDT Technologies (Supplementary Table 2). Samples were transferred to positively charged nylon membranes for 2 h and 15 min at 20 volts in 0.5X TBE buffer (45 mM Tris-borate, 1 mM EDTA). RNA was then fixed by UV radiation (120,000 μjoules) and membranes were blocked for 30 min at 41°C in pre-hybridization solution (6X SSC (150 mM NaCl, 15 mM Sodium Citrate), 5X Denhardt’s, 0.1% SDS, 100 μg/ml salmon sperm DNA). After blocking, probes were added directly to the hybridization solution (6X SSC, 0.1% SDS, 100 μg/ml salmon sperm DNA) and incubated overnight. Membranes were incubated for 3 min at room temperature with solution A (2X SSC, 0.1% SDS) and then incubated twice for 15 min at 41°C in solution B (0.1X SCC, 0.1% SDS). Next, membranes were blocked for 30 min at room temperature with blocking solution (1% casein in maleic buffer; 0.1 M maleic acid, 0.15 M NaCl pH 7.5). Then, 0.1 μg/ml of streptavidin- horseradish peroxidase was added to the blocking solution. Membranes were incubated for 30 min at room temperature and then cleaned twice for 15 min with maleic acid buffer 0.3% (v/v) tween-20. Finally, membranes were cleaned for 3 min in predetection buffer (0.1 M Tris–HCl, 0.1 M NaCl, pH 9.5) and developed using a chemiluminescent kit [SuperSignal West Pico Chemiluminescent Substrate (Thermo Scientific)].

### Determination of tRNA Aminoacylation Levels *in vivo*

Total RNA (obtained from the different strains that overexpress tRNA^*Gly*^ or tRNA^*Arg*^) was purified under acidic conditions and the 3′ extreme nucleotide was then eliminated by sodium periodate oxidation followed by β-elimination (Salazar et al., 2001; Choi et al., 2003). For this purpose, yeast was grown in EMM2 at 30°C, until OD600 of 0.9–1.0, and then pelleted at 10,000 × *g* for 6 min at room temperature. The pellet was resuspended in 500 μl of 0.3 M sodium acetate at pH 5.2, 1 mM of EDTA, followed by the addition of 500 μl of acid phenol. The mix was incubated for 10 min on ice and then centrifuged for 6 min at 10,000 × *g*. The supernatant was recovered, and RNA was precipitated adding three volumes of 100% ethanol at −80°C overnight. Samples were centrifuged for 30 min at 14,000 × *g* at 4°C. Pellets were cleaned with 0.5 ml of 75% ethanol, 10 mM sodium acetate pH 5.2 and then resuspended in 50 μl of water. Each sample was divided into two tubes (A and B), each with 25 μl aliquots. 1.42 μl of 3 M sodium acetate at pH 5.2 was added to the A tubes, and then stored at −80°C. tRNA in the B tubes was deacylated by adding 6.25 μl of 1 M Tris acetate at pH 9.0 and incubated for 60 min at 37°C. Samples in the B tubes were precipitated by adding 3.13 μl of 3 M sodium acetate at pH 5.2 and 62.5 μl of ethanol at 100%, and stored for 30 min at −80°C. Samples were centrifuged for 30 min at 13,000 × *g* at 4°C, pellets were washed with 70% ethanol and centrifuged at 13,000 × *g* for 5 min at 4°C. The pellet was dried and resuspended in 26.4 μl of 160 mM sodium acetate pH 5.2. Samples from the A tubes were thawed and 4.8 μl of freshly prepared 250 mM sodium periodate was added to each sample. Tubes were covered in aluminum paper and incubated for 90 min on ice, and then 12.97 μl of 20% glucose was added. After an additional 90 min of incubation on ice, 4.3 μl of 3 M sodium acetate at pH 5.2 and 87 μl of ethanol were added. Samples were stored for at least 30 min at −80°C and centrifuged for 30 min at13,000 × *g* at 4°C. Pellets were resuspended in 250 μl of 0.5 M lysine at pH 8.0 and incubated for 60 min at 45°C. Then, 25 μl of 3 M sodium acetate at pH 5.2 and 500 μl of ethanol were added and samples were stored for at least 30 min at −80°C. Tubes were centrifuged again for 30 min at 13,000 × *g* at 4°C and then washed with 70% ethanol. Finally, pellets were dried and resuspended in 15 μl of RNAse-free water. Samples were analyzed by gel electrophoresis (8 M urea, 10% polyacrylamide), followed by Northern blot analysis.

### mRNA Isolation and Quantification

To quantify *cdc13* mRNA, wild-type and over-expressed tRNA strains were grown to the late exponential phase (A600, 1.0) in 15 ml of EMM2 medium under standard conditions. Yeasts were then pelleted at 2,250 × *g* for 5 min at room temperature and washed once with sterilized water. Cell walls were disrupted with 0.12 μg of zymolyase 20T (United States Biological) in 1 M of sorbitol for 30 min at 37°C. Cells were pelleted and resuspended in 200 μl of TRIzol (Thermo Scientific) and vortexed three times for 1 min each (intercalated with 1 min on ice). Then, 40 μl of chloroform was added to the mix and immediately centrifuged at 12,000 × *g* for 20 min at 4°C. The supernatant was precipitated with 0.7 volumes of isopropanol at −80°C overnight. RNA was pelleted at 15,000 × *g* for 30 min and washed once with 80% ethanol. RNA was resuspended in 30 μl of sterilized miliQ water and quantified in a nanodrop spectrometer (BioTek), and then visualized in a 1% agarose gel to check RNA integrity. 1 μg of RNA was treated with DNAse I (Roche), according to the instructions provided in the manual. 500 ng of RNA was used for cDNA synthesis followed by Real-Time PCR with Brilliant II QRT-PCR, AffinityScript Two-Step Master Mix (Agilent), according to the manufacturer’s instructions. The primers used in the Real-Time PCR are listed in Supplementary Table 1 (cdc13rtF and cdc13rtR primers). Actin was used as a control in the quantifications.

### Cell-Cycle Synchronization by Hydroxyurea

Hydroxyurea (HU) was used to synchronize cells in the early-mid S phase, as described previously (Luche and Forsburg, 2009). Cells were grown in 5 ml of EMM2 at 30°C overnight, with constant agitation. Subsequently, the saturated culture was brought to an OD600 of 0.1 and incubated for 3 h or until an OD600 ∼0.2–0.6 HU was added at a final concentration of 15 mM and incubated for 4 h at 30°C with constant agitation. After the time, cells were collected by centrifuging at 4,000 × *g* for 5 min at room temperature. The supernatant was discarded, and the resulting sediment was washed twice with half the volume used of EMM2, previously incubated at 30°C, to remove residual HU. The sediment was resuspended in 1 ml of sterile EMM2 and incubated at 30°C with constant agitation, during all the experiment time (24 h) and a 10 μl aliquot was taken every 3 h. The phenotype was analyzed observing the cells by optical microscopy. A 40X objective (total magnification: 400) was used. The photos were obtained using a camera attached to a Canon SLR model Eos Rebel T3 lens microscopy.

### Total and Aggregated Protein Isolation and Quantification

Wild-type and mutant strains were grown at the exponential phase (OD600, 1.0) in 20 ml of EMM2 medium at 30°C with constant agitation. Yeasts were harvested by centrifugation at 2,250 × *g* for 5 min at room temperature, and the pellet was used to analyze protein aggregation as described previously (Rand and Grant, 2006). Briefly, cells were pelleted and resuspended in lysis buffer [50 mM potassium phosphate buffer, pH 7, 1 mM EDTA, 5% glycerol, 1 mM of phenylmethylsulfonyl fluoride, and Complete Mini protease inhibitor cocktail (Roche)]. Cell disruption was carried out by three vortex cycles (1 min of vortex and 1 min on ice) with 220 mg of acid-washed glass beads (Sigma-Aldrich G8772). Membrane proteins were removed by washing twice with 320 μl lysis buffer and 80 μl of 10% NP-40 (Sigma-Aldrich), and the final aggregated protein extract was resuspended in 20 μl of 1X Laemmli sample buffer. Total and aggregated protein extracts were analyzed by Western blot using an anti His-tag antibody in a 1:1,000 dilution (His Tag Antibody MAB050R-100, R&D Systems). Western blot against tubulin was performed as an internal control (T5168 monoclonal anti-a-tubulin clone B-5-1-2, Sigma-Aldrich). Bands representing WT, the pREP41_tRNA strain, and tubulin (for total protein), or WT and pREP41_tRNA strain (for aggregated proteins) were quantified using ImageJ software.

### Statistical Significance

The results are presented as mean ± standard deviations of the number of independent experiments indicated (biological replicates) (n) or as a representative example of experiments performed at least three times independently. Data were analyzed statistically using GraphPad Prism 6.0 software. The results were analyzed using the unpaired *t*-student test to determine significant differences among the experimental conditions. A *p* value < 0.05 was considered the limit of significance.

## Results

### Replacement of Rare Glycine Codons for Synonymous Preferred Codons in *cdc13* Severely Affects *S. pombe* Growth

**FIGURE 1:**
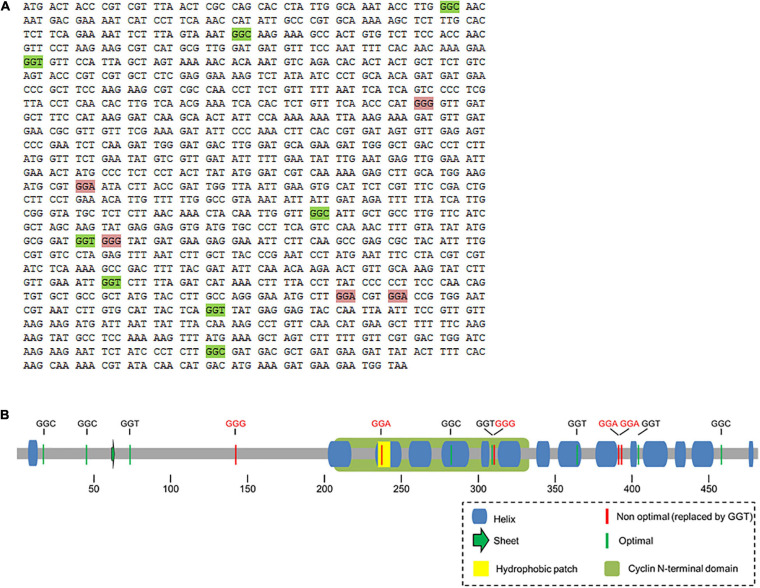
**(A)** Sequence of *cdc13* and localization of Gly codons. The coding sequence of *cdc13* is shown and unreplaced Gly codons (green boxes) and replaced codons (pink boxes) are indicated. **(B)** Schematic representation of secondary structure of Cdc13 from *S. pombe.* Location of glycine codons in the secondary structure of Cdc13 and the corresponding optimal (green) and non-optimal (red) codons in mRNA. The yellow square represents the hydrophobic motif MRGILTDW and the green rectangle represents the cyclin domain.

We used the gene encoding Cdc13, which is rich in non-optimal glycine codons to evaluate the effect of replacing non-optimal for optimal codons. GGA and GGG glycine codons are highly represented in *cdc13* (5 of the 13 glycine codons, 38.4% compared to less than 1% in highly-expressed genes) (Supplementary Table 3). Thus, we choose to replace these rare codons with the optimal GGT codon (86% of the glycine codons in highly-expressed genes) (Figure 1 and Supplementary Table 3), as described above. Surprisingly, the replacement of GGA/GGG codons with GGT in *cdc13*, yielded two types of colonies, small (most of them) and large (the remaining few) (Supplementary Figure 1, upper panel). The small colonies did not grow on either solid orin liquid media. The large colonies were cultured in liquid medium, where they grew slowly. Cell shape was observed by light microscopy at 16 h of culturing in rich medium. The mutant cells presented a phenotype similar to that of wild-type strains, although some cells were elongated, similar to what has been observed in yeast where the cell cycle is blocked after the G2 phase (Oltra et al., 2004; Supplementary Figure 1, lower panel). We believe that the replacement of rare glycine codons by optimal synonymous codons produced an altered Cdc13 protein that is non-functional to the cells, consequently cells stopped proliferating after a few duplications. Inspection of the *cdc13* sequence around the replaced codons did not reveal any alteration of off-frame stop codons (ambush hypothesis) (Seligmann and Pollock, 2004) that might account for the growth defect. However, large colonies proliferate even though *cdc13* contains all the rare glycine codon replacements (confirmed by DNA sequencing). The effect of replacing *cdc13* with mutated genes should be investigated, as should the differences in growth between the large and small colonies.

### tRNA^*Gly*^_*UCC*_ Overexpression Does Not Affect the Level of Other tRNA^*Gly*^

As noted above, tRNA levels in *S. cerevisiae* vary during the cell cycle, with the maximum at G2-M transition (Frenkel-Morgenstern et al., 2012). Since the replacement of rare glycine codons by optimal synonymous codons had a dramatic effect on *S. pombe* proliferation, we thought that the translation of rare codons may be controlled by the concentration of the decoding tRNA. The rare glycine codons GGA and GGG in *S. pombe* are decoded by the tRNA^*Gly*^_*UCC*_, encoded by three almost identical genes (low copy number compared to other tRNA^*Gly*^ genes). The tRNA^*Gly*^_*CCC*_ encoded by a single copy of the gene also decodes GGG codons (Supplementary Table 3).

We determined whether the expression of tRNA^*Gly*^_*UCC*_ is regulated in wild-type cells (as in *S. cerevisiae*) (Pang et al., 2014) by treating the cells with hydrogen peroxide and measuring the level of this tRNA. *S. pombe* cells were exposed to 5 or 10 mM of hydrogen peroxide and catalase activity was measured to confirm the response of the cells to oxidative stress. Increased catalase activity was observed under both conditions (data not shown). At 10 mM of hydrogen peroxide, the level of tRNA^*Gly*^_*UCC*_ increased fivefold, indicating that the level of tRNA^*Gly*^_*UCC*_ is regulated in response to this external challenge (Supplementary Figure 2A).

**FIGURE 2:**
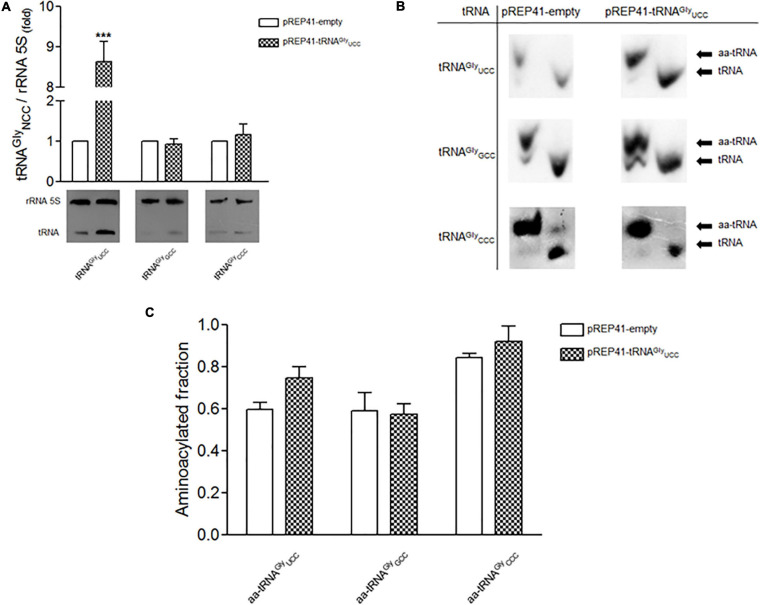
Overexpression of tRNA^*Gly*^_*UCC*_ (low gene dosage) and the effect on the level and aminoacylation of tRNA^*Gly*^ isoaceptors. **(A)** Lower panel. Northern blot of tRNA^*Gly*^_*UCC*_, tRNA^*Gly*^_*GCC*_ and tRNA^*Gly*^_*CCC*_ after overexpression of tRNA^*Gly*^_*UCC*_. Upper panel, relative levels of tRNA^*Gly*^ isoacceptors in cells that overexpress tRNA^*Gly*^_*UCC*_. 5S rRNA was used as a reference (folds of change (***p < 0,001, *t*-student). **(B)** Aminoacylation of tRNA^*Gly*^ isoacceptors. Representative Northern blot analysis of total tRNA purified under acidic conditions from *S. pombe* transformed with pREP41-empty (control) or pREP41-tRNA^*Gly*^_*UCC*_, detected with specific probes against tRNA^*Gly*^_*UCC*_, tRNA^*Gly*^_*GCC*_, and tRNA^*Gly*^_*CCC*_. Aminoacylated tRNAs (aa-tRNA) and non-aminoacylated tRNAs (tRNA) are indicated. **(C)** Quantification of Northern blots. Relative levels of aminoacylated fraction of tRNA^*Gly*^_*UCC*_, tRNA^*Gly*^_*GCC*_, and tRNA^*Gly*^_*CCC*_ for each condition, were quantified by densitometric analysis.

To test whether an increase in tRNA^*Gly*^_*UCC*_ concentration affects the level of Cdc13, we overexpressed this tRNA in *S. pombe*. For this purpose, we cloned the corresponding tRNA gene (which contains an internal RNA polymerase III promoter) in the high copy number vector pREP41, with LEU2 as selection marker. *S. pombe* cells were transformed with this construct (Supplementary Figure 2B), with the empty vector as a control. Northern blot analysis using a specific probe for tRNA^*Gly*^_*UCC*_ revealed a 7-to-8-fold increase in the level of this tRNA compared to the control (Figure 2A). The overexpression of tRNA^*Gly*^_*UCC*_ did not affect the levels of other tRNA^*Gly*^ (tRNA^*Gly*^_*GCC*_, tRNA^*Gly*^_*CCC*_) (Figure 2A). We also tested the aminoacylation status of these tRNA^*Gly*^ with overexpression of tRNA^*Gly*^_*UCC*_. The ratio of aminoacylated/deacylated tRNA in cells transformed with the empty vector and cells that overexpressed tRNA^*Gly*^_*UCC*_ was determined. We observed a ∼20% increase in the amino acylated fraction of tRNA^*Gly*^_*UCC*_ compared to the control (Figures 2B,C) (although it was not a statistically significant) The fact that the level of this tRNA increased 7-to-8-fold in cells transformed with the vector containing the tRNA^*Gly*^_*UCC*_ gene (Figure 2A) implies that the amount of Gly-tRNA^*Gly*^ increases 10-to-11-fold compared to the control. However, the overexpression of tRNA^*Gly*^_*UCC*_ did not significantly change the amino acylation levels of other tested tRNA^*Gly*^ isoacceptors (Figures 2B,C).

### Overexpression of tRNA^*Gly*^_*UCC*_ Affects the Distribution of Cdc13 Into Soluble and Aggregated Fractions

**FIGURE 3:**
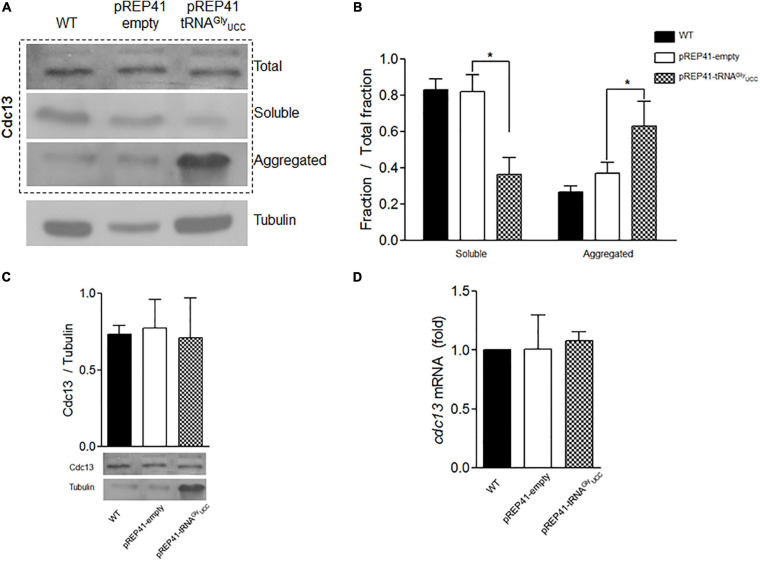
Overexpression of tRNA^*Gly*^_*UCC*_ in *S. pombe* affects the aggregation status of Cdc13. **(A)** Western blot of Cdc13 in total extract or soluble and aggregated fractions. **(B)** Quantification of soluble and aggregated Cdc13 compared to total extract. **(C)** Total Cdc13 in cells that overexpress tRNA^*Gly*^_*UCC*_ compared to controls. **(D)** Relative levels of *cdc13* mRNA compared to those of controls (pREP41-empty). (*p < 0.05, *t*-student).

We used cells transformed with pREP4-tRNA^*Gly*^_*UCC*_ and control cells to evaluate the effect of tRNA^*Gly*^_*UCC*_ overexpression on Cdc13 expression. Neither total Cdc13 protein levels (Figures 3A–C) nor its mRNA levels (Figure 3D) were altered by tRNA^*Gly*^_*UCC*_ overexpression. However, when the soluble and aggregated fractions were separated, a dramatic increase in Cdc13 was observed in the aggregated fraction (Figure 3A, quantified in Figure 3B). Together, these results suggest that increasing tRNA^*Gly*^_*UCC*_ levels increases the Cdc13 in the aggregated fraction without affecting its level. Further investigation is needed to determine if this is the result of Cdc13 misfolding because of a change in the mRNA translation rate. An alternative explanation for this defect is that other proteins (including chaperones) are altered by tRNA^*Gly*^_*UCC*_ overexpression and aggregate with Cdc13. Another explanation is that tRNA^*Gly*^_*UCC*_ overexpression causes increased mismatches with non-cognate codons that are not compensated (Seligmann, 2011), giving rise to translation errors that induce protein aggregations. These and other alternative explanation need to be investigated.

### Overexpression of tRNA^*Gly*^_*UCC*_ Affects the Progression of the *S. pombe* Cell Cycle

**FIGURE 4:**
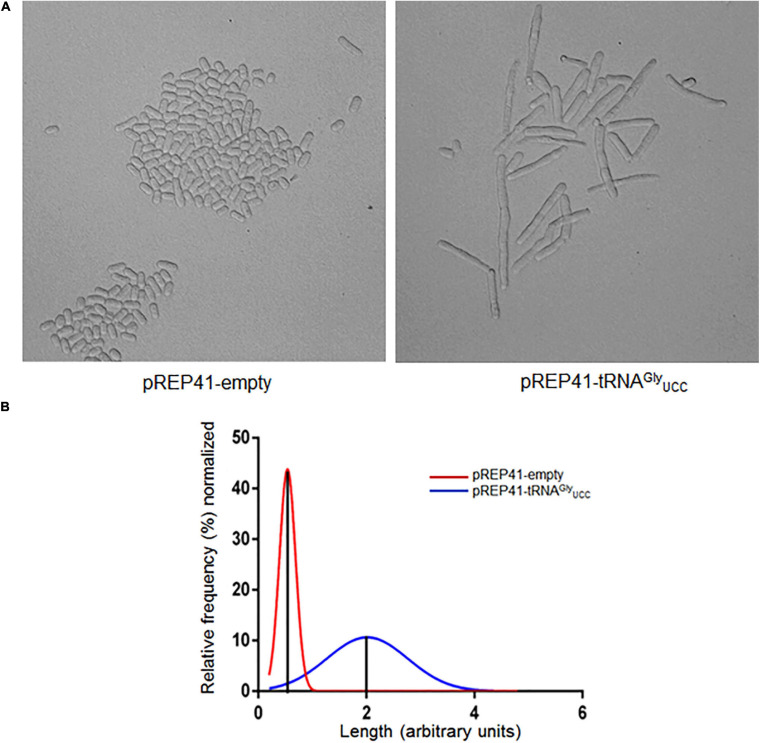
Overexpression of tRNA^*Gly*^_*UCC*_ causes morphological changes, inducing cell elongation. **(A)** Phenotype observed under a microscope of cells synchronized with 15 mM HU for 4 hrs. The images were obtained under a microscope at 400x at 12 h post-incubation with HU (cell cycle stage: G2). **(B)** Histograms of the length of cells that overexpress tRNA^*Gly*^_*UCC*_ (blue line) normalized by the average length of control cells (red line).

To test whether the overexpression of tRNA^*Gly*^ affects the cell cycle progression of *S. pombe*, yeast cultures were synchronized with HU. The shapes of cells that overexpress tRNA^*Gly*^_*UCC*_ and control cells were monitored under a microscope at 12 h (three cell cycles). Under these conditions, we observed that nearly 100% of cells that overexpress tRNA^*Gly*^_*UCC*_ had elongated shapes (Figure 4A). The average size of cells that overexpress tRNA^*Gly*^_*UCC*_ was at least twice that of the control cells, but some were four times as large (Figure 4B). More than one septum was observed microscopically in the elongated cells. Unsynchronized cells also evidenced elongated shape, but to a lesser extent.

**FIGURE 5:**
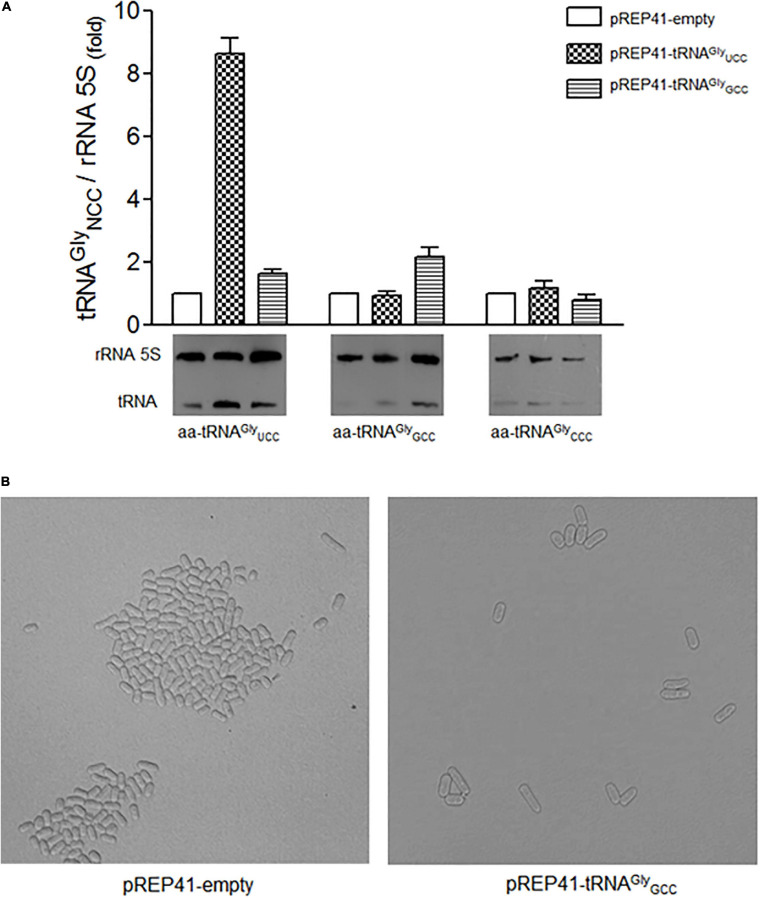
Overexpression of tRNA^*Gly*^_*GCC*_ did not change the level of other tRNA^*Gly*^ isoaceptors and did not cause morphological changes in cell elongation. **(A)** Relative levels of tRNA^*Gly*^_*UCC*,_ tRNA^*Gly*^_*GCC*_, tRNA^*Gly*^_*CCC*_ isoacceptors in cells transformed with the indicated constructs. **(B)** Phenotype observed under a microscope of cells overexpressing tRNA^*Gly*^_*GCC*_ synchronized with 15 mM HU for 4 hrs. The images were obtained under a microscope at 400x at 12 hrs post-incubation with HU (cell cycle stage: G2).

In order to evaluate whether the elongated phenotype is a specific result of the overexpression of tRNA^*Gly*^_*UCC*_ and not the overexpression of any other tRNA^*Gly*^, we overexpressed the high copy number tRNA^*Gly*^_*GCC*_ gene in *S. pombe* that decodes both optimal glycine codons. We observed a twofold increase in tRNA^*Gly*^_*GCC*_ (Figure 5A). As tRNA^*Gly*^_*GCC*_ is encoded by eight copies (Supplementary Table 3), we expected relatively high levels of this tRNA in the cells. However, *S. pombe* showed no elongated phenotypes (Figure 5B). Similar experiments involved overexpressing tRNA^*Arg*^_*UCU*_. This tRNA decodes the arginine AGA (preferred) and AGG (rare) codons present in *S. pombe cdc13*. The tRNA gene was cloned as indicated for tRNA^*Gly*^_*UCC*_. A 2.5-fold increase in the cellular level of tRNA was observed (data not shown). However, the overexpression of tRNA^*Arg*^_*UCU*_ did not alter cell shape, as tRNA^*Gly*^_*UCC*_ overexpression did (data not shown).

## Discussion

Translation efficiency and accuracy are largely achieved by binding to the ribosome of the proper aminoacyl-tRNA that competes with a plethora of non-cognate or near-cognate aminoacyl-tRNAs (all at different concentrations) to enter the A site, Ikemura (1985), Kanaya et al. (1999), Frenkel-Morgenstern et al. (2012), Kirchner et al. (2017), Torrent et al. (2018), Yang et al. (2020). This gives rise to the notion that optimal codons are translated by highly represented transfer RNAs. The translation speed of certain codons has been explained as the result of the time required by the ribosome to find the proper aminoacyl-tRNA to translate the codon in the A site. Rare codons are usually translated by lowly-represented tRNAs. The ribosome must deal with stochastic binding of these tRNAs in competition with the entire pool of highly represented tRNAs, which slows down the translation of rare codons. There are a few examples where the level of transfer RNAs alters or regulates the translation of genes crucial for cellular processes based on the presence of non-optimal codons, in particular mRNAs. A mutation that replaces an optimal codon by a rare synonymous codon in human CFTR gene lead to a misfolded and malfunctioning proteins. An increase in the level of tRNA decoding such a codon restores the function of CFTR (Kirchner et al., 2017). In yeast, stress-responsive genes are enriched in codons that use rare tRNAs. The tRNAs of cells exposed to different stresses are reprogrammed to respond to stress by enhancing stress mRNA translation (Torrent et al., 2018). Certain tRNAs are preferentially expressed in human cancer cells under the control of an RNA polymerase III transcription factor. The knockdown of these tRNAs reduces the proliferation of cancer cells, which indicates their crucial role in the reprogramming cell proliferation (Yang et al., 2020).

The cell cycle is a complex process that requires the temporal expression of a number of proteins that regulate the functioning of the different phases of the process in coordination. The fission yeast *S. pombe* has been a model to study the cell cycle of eukaryotes (Yanagida, 2002). Many of the cell cycle proteins from *S. pombe* are temporarily expressed based on the transcription and translation of corresponding genes, as well as the degradation of gene products in a well-coordinated process (Hayles et al., 2013). Several of these proteins are enriched in rare codons, giving rise to the notion that translation efficiency is in part a way to control the level of some proteins. The levels of transfer RNAs oscillate markedly, with increases in the G2 phase, concomitant with an increase in the activity of several amino acyl tRNA synthetases, including GlyRS (Frenkel-Morgenstern et al., 2012). The two molecular events match with the expression patterns of certain cyclins. These observations reinforce the idea that translation of these proteins is regulated cyclically by tRNA levels.

Cdc13 is a crucial protein that controls the G2-M transition of the *S. pombe* cell cycle. The presence of five non-optimal codons of the thirteen codons for glycine in *cdc13* suggests that the level of this protein is controlled at the translational level. The data obtained in this work reveals that alterations of rare codons decoding glycine in the gene that encodes Cdc13 have a profound impact on cell proliferation. First, the replacement of all five codons by optimal counterparts, where 2 of the 5 rare glycine codons are in the cyclin N-terminal domain (Figure 1), and the other two are located in tandem near the C-terminus, results in the almost complete impairment of the cells to proliferate in solid or liquid media (Supplementary Figure 1). It is believed that synonymous codons tend to be translated more slowly than optimal counterparts (Shah and Gilchrist, 2011; Boël et al., 2016) suggesting an alteration of co-translational protein folding. Other reports suggest that protein synthesis is under selective evolutionary pressure by co-translational folding (Chaney et al., 2017; Jacobs and Shakhnovich, 2017). One hypothesis is that subtle modifications in the elongation rate affect the folding mechanism (Braselmann et al., 2013), although cells have molecular chaperones that help in folding proteins adequately, including folding nascent polypeptides (Kramer et al., 2019). According to our results it is possible that the replacement of rare glycine codons by optimal counterparts results in misfolded Cdc13, as has been suggested (Zhou et al., 2015), This would reduce the levels of functional Cdc13, although there may be other interpretations of the results (see below).

Studies have shown that a complex between Cdc2 (Cdk1) and Cdc13 is required for the cells in *S. pombe* to enter mitosis. It has been reported that the deletion of the *cdc13* gene gives rise to small cells that do not enter mitosis, although some of them can continuously replicate, giving rise to elongated cells with giant nuclei (Hayles et al., 1994). Patterson et al. (2019) described another example of elongated cells and demonstrated that Cdc13 expression below wild-type levels results in larger cells. They found a correlation between Cdc13 expression levels and cell size at division. Using mutant cells with a thermosensitive mutation in *cdc13*, they found that at the restrictive temperature, the complex was largely in the insoluble fraction, which prevents the cell from entering mitosis (Hayles et al., 1994). Our results show that overexpression of tRNA^*Gly*^_*UCC*_ is accompanied by a substantial increase in Cdc13 in the aggregated protein fraction (up to 50% of the all Cdc13 proteins, Figure 3), along with the formation of elongated cells (Figure 4). The effects observed in our work may be the result of several different events, such as aggregation of other proteins that drag Cdc13, impairment of chaperones to properly fold Cdc13, or effects on other cyclins. However, the observed effects are consistent with the role of Cdc13 in forming the complex described above (Hayles et al., 1994; Humaidan et al., 2018) and the cell replication problem as a consequence of replacing rare Gly codons in *cdc13.* Further experiments are required to confirm whether there is a direct effect on Cdc13 folding and aggregation that alters the cell cycle. Nevertheless, these two effects seem to be specific to tRNA^*Gly*^_*UCC*_, as they are not observed when the tRNAs that decode optimal glycine or arginine codons are overexpressed.

The overexpression of Gly-tRNA^*Gly*^_*UCC*_ probably exerts an effect on not only *cdc13* mRNA, but also many other mRNAs containing the decoded codons. Thus, the observed cell division phenotype maybe the consequence of the altered expression of other genes involved in the cell cycle. Analysis of the effect of global proteome alterations on tRNA^*Gly*^_*UUC*_ overexpression, or the overexpression of any other tRNAs will certainly give insights into the role of rare codons in the cell cycle, as well as the selective pressure that allows the natural selection of rare codons in cell cycle proteins.

## Data Availability Statement

The original contributions presented in the study are included in the article/Supplementary Material, further inquiries can be directed to the corresponding author/s.

## Author Contributions

LA conducted most of the experiments as part of her Magister thesis. FM, DG, and RF-R contributed with part of the experimental data. MT conducted all bioinformatic analysis. SM supervised the thesis of LA and contributed with her expertise on *S. pombe* manipulation. AK contributed with ongoing discussion and reviewing the manuscript. OO provided the funds, conceptual questions, ongoing discussion and training of students. All authors contributed to the article and approved the submitted version.

## Conflict of Interest

The authors declare that the research was conducted in the absence of any commercial or financial relationships that could be construed as a potential conflict of interest.
