# Supplementary material for: Modification of Transfer RNA Levels Affects Cyclin Aggregation and the Correct Duplication of Yeast Cells
Source: Front Microbiol. 2021 Jan 15;11:607693. doi: 10.3389/fmicb.2020.607693 (PMC7843576; doi:10.3389/fmicb.2020.607693)
Supplement: Supplementary file 1 [file Data_Sheet_1.DOCX]

**Supplementary material**

Table 1. **Primers used in this study**

| **Primers** | | **Sequence (5'-… -3)** |
| --- | --- | --- |
| cdc13-histag | forward | GGAATTCCATATGACTACCCGTCGTTTAACT |
| cdc13-histag | reverse | CGGGATCCTTAATGATGGTGATGATGGTGACCGCTACCCATCCATTCTTCATCTTTCAT |
| cdc13F | forward | CGAGCTCGTATTGTGCATATCACTATCTCAACCT |
| cdc13R | reverse | CCGGAATTCATAATCAAATCTAAGACTTAAA |
| Pfa6aF1 | forward | GAAGTCTCAAAAGCTCTTGCC |
| PFA6AF2 | forward | GCTTCTGTCAGTACCCGTCG |
| pFA6aR | reverse | GCTTCGCGCCGTGCGGCCAT |
| tRNAglyUCCF' | forward | GGGAATTCCATATGCAGTAAATCAATAAACCAAA |
| tRNAglyUCCR' | reverse | CGCGGATCCACTAGCAACTGCTAAATAAA |
| tRNAargUCU_IIF' | forward | GGGAATTCCATATGCGGTTGACATTTCAATAAAAA |
| tRNAargUCU_IIR' | reverse | CGCGGATCCCATCCTCAGGATTTCAAGTT |
| tRNAglyGCCF' | forward | GGGAATTCCATATGGCTTTACAGCTTAATTCGAAG |
| tRNAglyGCCR' | reverse | CGCGGATCCCAGAATGCTTGTAGAATCTCGC |
| pREP41F' | forward | GCTGTAAAACACCACGAGAC |
| pREP41R' | reverse | CTCGTTGTCGGAGATCAAGA |
| cdc13rtF | forward | TAAGAAGCGTCATGCGTTGG |
| cdc13rtR | reverse | TGGAAGCGGGTTCATCATCT |
| ActinaF' | forward | CGGTCGTGACTTGACTGACT |
| ActinaR' | reverse | TCAAGGGAGGAAGATTGAGC |
| CDC13_GGT1_F | forward | TCAACCCATGGTGTTGATGCTTTC |
| CDC13_GGT1_R | reverse | GAAAGCATCAACACCATGGGTTGA |
| CDC13_GGT2_F | forward | ATGCGTGGTATACTTACCGATTGG |
| CDC13_GGT2_R | reverse | CCAATCGGTAAGTATACCACGCAT |
| CDC13_GGT3_F | forward | GATGGTGGTTATGATGAAGAGG |
| CDC13_GGT3_R | reverse | CCTCTTCATCATAACCACCATC-3 |
| CDC13_GGT45_F | forward | AGGGAAATGCTTGGTCGTGGTCCGTGG |
| CDC13_GGT45_R | reverse | CCACGGACCACGACCAAGCATTTCCCT |

**Table 2. Oligonucleotides used for tRNA quantification.**

| **Probe** | **Sequence (5'-...-3')** |
| --- | --- |
| tRNA^Gly^_UCC_ | TGGAAGGCATTGATGTTACCGCTA |
| tRNA^Arg^_UCU_ | ATTAGAAGTGAGATGCTCTACCATTGA |
|  | ATTAGAAGTCAAATGCCCTAGCCATTA |
| tRNA^Gly^_GCC_ | GATGGCAACGATTCATTATACCACTA |
| tRNA^Gly^_CCC_ | CATGGGAAGCTCCAATGTTACCGCTA |
| rRNA 5S | CTAACGAGGCCCTCAGACGCTTAACTGCAGT |
|  |  |

**Table 3. Copy number of tRNA^Gly^ issoaceptor genes in *S. pombe* genome and decoding codons (Forsburg, S. 1994)**

| **tRNA** | **Number of copies** | **Decoded codon** |
| --- | --- | --- |
|  |  | % usage codon (3 high expressed genes on *S. pombe*) |
| **Gly_UCC_** | **3** | GGA/GGG |
|  | (low gene dosage) | 0% (rare codons) |
| **Gly_GCC_** | 8 | GGC/GGT |
|  | (high gene dosage) | 14% / 86% (optimal codons) |
| **Gly_CCC_** | 1 | GGG |
|  | (low gene dosage) | 0% (rare codon) |

**Supplemetary Figures.**

.


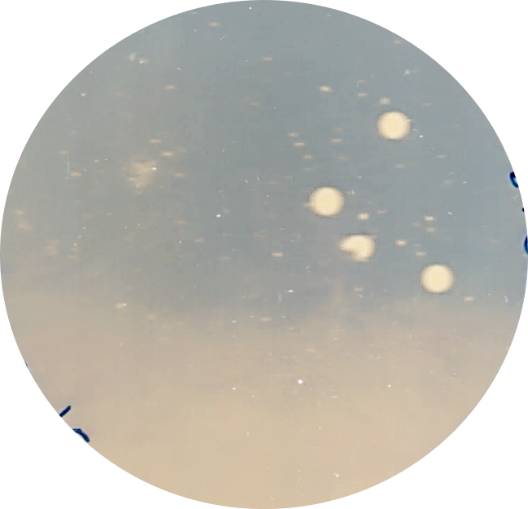

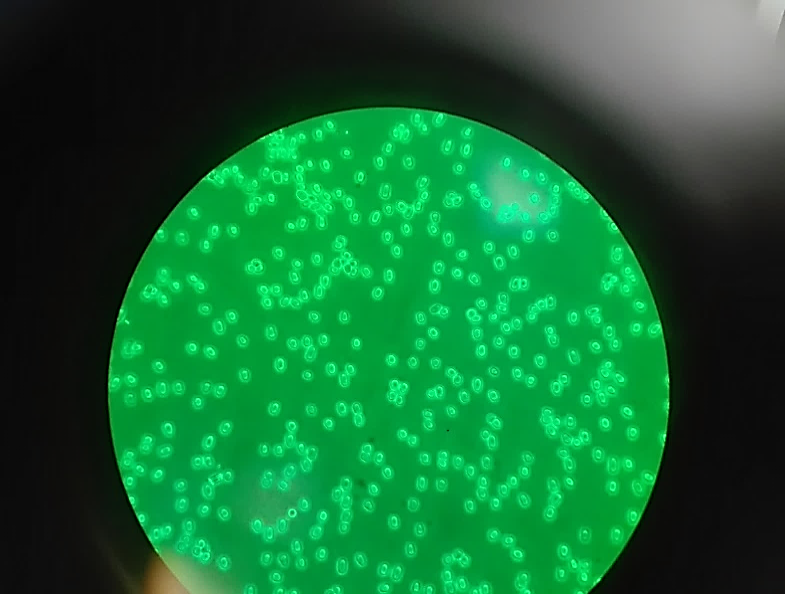


**LP36**


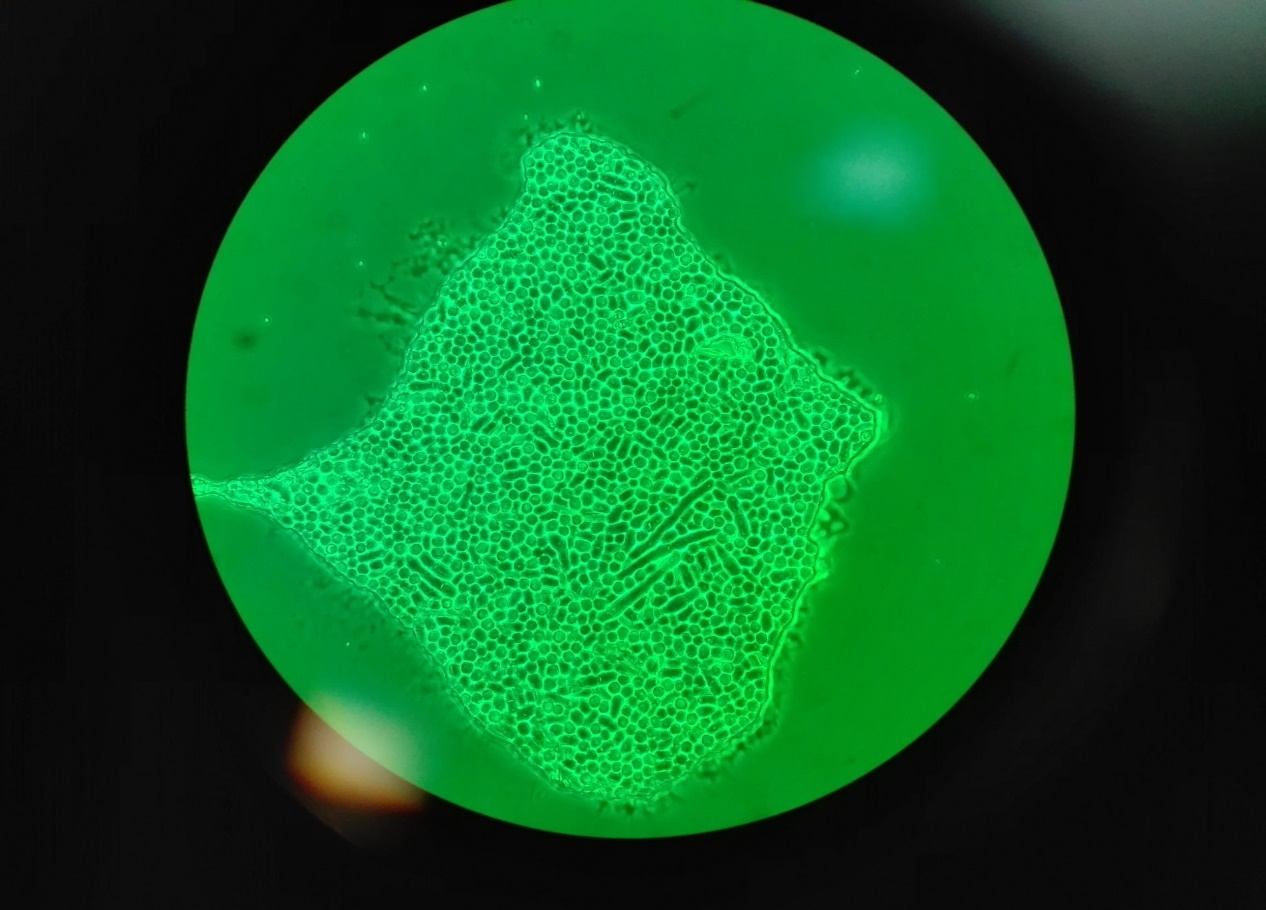


**Cdc13_mut**

**Supplementary Figure 1**. Growth of *S. pombe* with mutated *cdc13.* Rare Gly codons were replaced by preferred codons in *cdc13.* Wild type gene was replaced by the mutated gene and cells were grown in solid medium. Upper pannel, growth of cells with mutated gene in solid medium. Red square shows an example of small colonies (majority) and green square the few large colonies. Lower pannel, Microscopic observation of colonies of wild type (LP36) as control and large colony of cells with mutated *cdc13* (Cdc13_mut).

**A.**


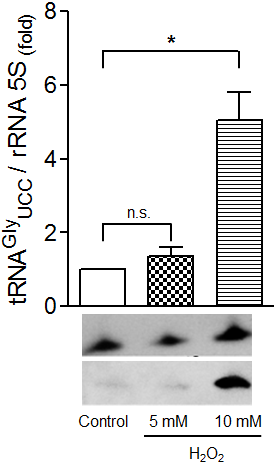


**B.**

**
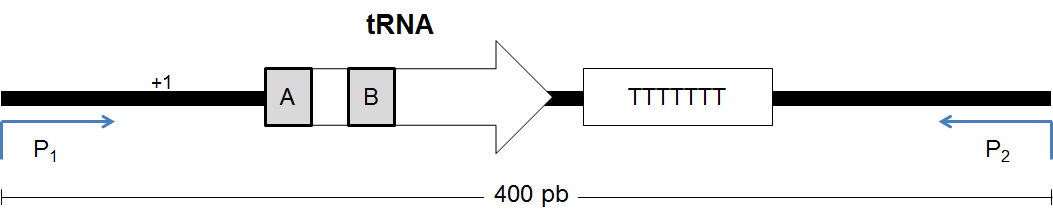
**

**Supplementary Figure 2. tRNA^Gly^_UCC_ levels in *S. pombe* under oxidative stress condition**. A. Lower pannel. Northern blot analysis of tRNA^Gly^_UCC_. 5S rRNA was used as internal control. Upper pannel. Relative levels of tRNA^Gly^_UCC_ at 5 and 10 mM H_2_O_2_ compared to untreated control cells. (* p<0.05, t-Student). B. Schematic representation of the plasmid pREP41 containing the tRNA genes. Boxes A and B represent the internal transcription promoter elements, +1 the transcription initiation and the stretch of Ts the transcripcion terminator. P_1_ and P_2_ the primers used for amplification of the tRNA genes from the genomic DNA of *S. pombe.*
